# On the impact of uncertain gene tree rooting on duplication-transfer-loss reconciliation

**DOI:** 10.1186/s12859-018-2269-0

**Published:** 2018-08-13

**Authors:** Soumya Kundu, Mukul S. Bansal

**Affiliations:** 1 Department of Computer Science and Engineering, University of Connecticut, Storrs, CT, 06269 USA; 2 Institute for Systems Genomics, University of Connecticut, Storrs, CT, 06269 USA

**Keywords:** Phylogenetics, Reconciliation, Gene trees, Horizontal gene transfer, Microbial evolution

## Abstract

**Background:**

Duplication-Transfer-Loss (DTL) reconciliation is a powerful and increasingly popular technique for studying the evolution of microbial gene families. DTL reconciliation requires the use of rooted gene trees to perform the reconciliation with the species tree, and the standard technique for rooting gene trees is to assign a root that results in the minimum reconciliation cost across all rootings of that gene tree. However, even though it is well understood that many gene trees have multiple optimal roots, only a single optimal root is randomly chosen to create the rooted gene tree and perform the reconciliation. This remains an important overlooked and unaddressed problem in DTL reconciliation, leading to incorrect evolutionary inferences. In this work, we perform an in-depth analysis of the impact of uncertain gene tree rooting on the computed DTL reconciliation and provide the first computational tools to quantify and negate the impact of gene tree rooting uncertainty on DTL reconciliation.

**Results:**

Our analysis of a large data set of over 4500 gene families from 100 species shows that a large fraction of gene trees have multiple optimal rootings, that these multiple roots often, but not always, appear closely clustered together in the same region of the gene tree, that many aspects of the reconciliation remain conserved across the multiple rootings, that gene tree error has a profound impact on the prevalence and structure of multiple optimal rootings, and that there are specific interesting patterns in the reconciliation of those gene trees that have multiple optimal roots.

**Conclusions:**

Our results show that unrooted gene trees can be meaningfully reconciled and high-quality evolutionary information can be obtained from them even after accounting for multiple optimal rootings. In addition, the techniques and tools introduced in this paper make it possible to systematically avoid incorrect evolutionary inferences caused by incorrect or uncertain gene tree rooting. These tools have been implemented in the phylogenetic reconciliation software package RANGER-DTL 2.0, freely available from http://compbio.engr.uconn.edu/software/RANGER-DTL/.

## Background

Duplication-Transfer-Loss (DTL) reconciliation is one of the most effective techniques for studying the evolution of gene families and inferring evolutionary events such as gene duplications, horizontal gene transfers, and gene losses. Given the evolutionary tree for a gene family, i.e., a *gene tree*, and the evolutionary tree for the corresponding species, i.e., a *species tree*, DTL reconciliation compares the gene tree with the species tree and reconciles any differences between the two by proposing gene duplication, horizontal gene transfer, and gene loss events. Accurate knowledge of these events and of gene family evolution overall has many important applications throughout biology, and the DTL reconciliation problem has therefore been extensively studied, e.g., [1–13].

DTL reconciliations are generally computed using a parsimony framework where each evolutionary event is assigned a cost and the goal is to find a reconciliation with minimum total cost. The resulting optimization problem is called the *DTL-reconciliation problem*. Computed DTL reconciliations can sometimes be *time-inconsistent*; i.e, the inferred transfers may induce contradictory constraints on the dates for the internal nodes of the species tree. The problem of finding an optimal *time-consistent* reconciliation is known to be NP-hard [3, 14]. In practice, there are two standard formulations of the DTL-reconciliation problem. In the first formulation, the goal is to find an optimal (not necessarily time-consistent) DTL reconciliation [3–5, 8, 10]; this is computable in *O*(*mn*) time [5], where *m* and *n* denote the number of nodes in the gene tree and species tree, respectively. The second standard formulation is based on the observation that the problem of finding an optimal time-consistent reconciliation becomes efficiently solvable [2, 15] in *O*(*mn*^2^) time if the species tree is fully dated, and thus requires the use of a fully dated species tree [2, 9]. However, accurately dating the internal nodes of a species tree is notoriously difficult [16]. Consequently, in this work, we focus primarily on the first (undated species tree) formulation of the problem, though we also study the effect of using dated species trees.

Both of the standard formulations of the DTL-reconciliation problem require the gene tree and the species tree to be rooted. However, while species trees can generally be confidently rooted (using outgroups, for example), gene trees are often difficult to root. As a result, the gene trees used for DTL reconciliation are often unrooted. When provided with an unrooted gene tree, existing DTL reconciliation algorithms and software first find a root for the unrooted gene tree and then use the resulting rooted gene tree for the reconciliation. The approach employed for rooting unrooted gene trees is to compute the reconciliation cost for each possible rooting of the unrooted gene tree and then choose a rooting that yields the minimum reconciliation cost. There is, however, a critical flaw in this approach: Many gene trees have multiple optimal roots, yet only a single optimal root is randomly chosen to create the rooted gene tree and perform the reconciliation. This is one of the most important unaddressed problems in DTL reconciliation, with direct bearing on the accuracy of the inferred reconciliation.

**Previous work.** The problem of multiple optimal roots has been largely overlooked in DTL reconciliation literature. A recent paper by Urbini et al. [17] studied the effect of rooting uncertainty on reconciliation in the context of host-symbiont cophylogeny. Host-symbiont cophylogeny reconciliation is similar (though not identical) to DTL reconciliation, so the results of their study are also of relevance to DTL reconciliation. They applied host-symbiont cophylogeny reconciliation to several small data sets and measured the impact of alternative rootings on the number of inferred evolutionary events (but not on the reconciliation itself). They also established that host-symbiont cophylogeny reconciliations need not satisfy the “plateau” property, in which all optimal roots must appear clustered together in a particular fashion on the gene tree. This plateau property is known to hold for some simpler phylogenetic reconciliation models that do not handle horizontal gene transfers [18]. Thus, there is currently little insight into the prevalence and patterns of multiple optimal rooting in large biological data sets, almost no understanding of how DTL reconciliations change across different optimal rootings, and no techniques or tools to systematically account for reconciliation uncertainty due to multiple optimal roots.

**Our contributions.** Here, we perform the first in-depth analysis of the impact of uncertain gene tree rooting on DTL reconciliation and provide the first computational tools to quantify and negate the impact of gene tree rooting uncertainty. We analyze a large data set of over 4500 gene families from 100 species and (i) show that a large fraction of gene trees have multiple optimal rootings, (ii) show that these multiple roots often, but not always, appear clustered together in the same region of the gene tree, (iii) define the notion of a *consensus reconciliation* which captures the variability in the reconciliation due to multiple gene tree rootings, (iv) compute consensus reconciliations and use them to show that many aspects of the reconciliation remain conserved across the multiple rootings, and (v) demonstrate that gene tree error has a profound impact on the prevalence and structure of multiple optimal rootings. We also show that there are specific interesting patterns in the reconciliations of singly rooted and multiply rooted gene trees. Our analysis also considers the influence of different event cost assignments and of using dated species trees.

The techniques and tools introduced in this paper make it possible to systematically avoid incorrect evolutionary inferences caused by incorrect or uncertain gene tree rooting. Our tools for computing consensus reconciliations have been implemented in the phylogenetic reconciliation software package RANGER-DTL 2.0, freely available from http://compbio.engr.uconn.edu/software/RANGER-DTL/.

## Methods

### Definitions and preliminaries

#### Basic definitions

We follow the basic definitions and notation from [5]. Given a tree *T*, we denote its node, edge, and leaf sets by *V*(*T*), *E*(*T*), and *Le*(*T*), respectively.

If *T* is rooted, the root node of *T* is denoted by *rt*(*T*), the parent of a node *v*∈*V*(*T*) by *pa*_*T*_(*v*), its set of children by *Ch*_*T*_(*v*), and the (maximal) subtree of *T* rooted at *v* by *T*(*v*). The set of *internal nodes* of *T*, denoted *I*(*T*), is defined to be *V*(*T*)∖*Le*(*T*). For a rooted tree *T*, we define ≤_*T*_ to be the partial order on *V*(*T*) where *x*≤_*T*_*y* if *y* is a node on the path between *rt*(*T*) and *x*. The partial order ≥_*T*_ is defined analogously, i.e., *x*≥_*T*_*y* if *x* is a node on the path between *rt*(*T*) and *y*. We say that *y* is an *ancestor* of *x*, or that *x* is a *descendant* of *y*, if *x*≤_*T*_*y* (note that, under this definition, every node is a descendant as well as an ancestor of itself). For each node *v*∈*I*(*T*), the *cluster* *C*_*T*_(*v*) is defined to be the set of all leaf nodes in *T*_*v*_; i.e. *C*_*T*_(*v*)=*Le*(*T*_*v*_). We denote the set of all clusters of a tree *T* by *Cluster*(*T*). A tree is *binary* if all of its internal nodes have exactly two children. Throughout this work, the term *tree* refers to binary trees.

If *T* is unrooted, then there are exactly |*E*(*T*)| different ways of rooting *T* (by adding a root node on an edge). Let *Root*(*T*) denote the set of rooted trees that can be obtained by rooting *T*.

We denote the gene tree and species tree under consideration by *G* and *S*, respectively. If *G* is unrooted, we refer to it as *G*^*U*^, and as *G*^*R*^ if it is rooted. We assume that each leaf of the gene tree is labeled with the species from which that gene was sampled. This labeling defines a *leaf-mapping*$\mathcal {L}_{G,S}\colon {Le}(G) \rightarrow {Le}(S)$ that maps a leaf node *g*∈*Le*(*G*) to that unique leaf node *s*∈*Le*(*S*) which has the same label as *g*. Note that gene trees may have more than one gene sampled from the same species. We implicitly assume that the species tree contains all the species represented in the gene tree.

#### DTL reconciliation

A rooted gene tree can be reconciled with a rooted species tree by mapping the gene tree onto the species tree and labeling each gene tree node as representing either a speciation, duplication, or transfer event. Any DTL reconciliation for *G*^*R*^ and *S* shows a possible evolutionary history of the gene inside the species tree. To be a biologically valid evolutionary history, the reconciliation must satisfy certain constraints on the mapping of *G*^*R*^ onto *S*. For further details on these constraints, we refer the reader to the definition of *DTL-scenario* from [3, 5]. Essentially, any valid DTL-scenario maps each gene tree node to a unique species tree node in a consistent way that respects the immediate temporal constraints implied by the species tree and designates each gene tree node as representing either a speciation, duplication, or transfer event. More precisely, any DTL scenario for *G*^*R*^ and *S* partitions *I*(*G*^*R*^) into the sets *Σ*, *Δ*, and *Θ* representing speciation, duplication, and transfer events, respectively, and specifies a mapping ${\mathcal {M}} \colon V\left ({G^{R}}\right) \rightarrow V(S)$ that maps each node of *G*^*R*^ to a node of *S*.

DTL-scenarios correspond naturally to reconciliations and it is straightforward to infer the reconciliation of *G*^*R*^ and *S* implied by any DTL-scenario.

Given a DTL-scenario *α*, one can directly count the minimum number of gene losses, *Loss*_*α*_, in the corresponding reconciliation. For brevity, we refer the reader to [5] for further details on how to count losses in DTL-scenarios.

Let *P*_*Δ*_, *P*_*Θ*_, and *P*_*loss*_ denote the non-negative costs associated with duplication, transfer, and loss events, respectively.

##### **Definition 1**

(Reconciliation cost of a DTL-scenario) Given a DTL-scenario *α* for *G*^*R*^ and *S*, the *reconciliation cost* associated with *α* is given by ${\mathcal {R}}_{\alpha } = P_{\Delta } \cdot |\Delta | + P_{\Theta } \cdot |\Theta | + P_{loss} \cdot {Loss}_{\alpha }$.

A most parsimonious reconciliation is one that has minimum reconciliation cost.

##### **Definition 2**

(Most Parsimonious Reconciliation (MPR)) Given *G*^*R*^ and *S*, along with *P*_*Δ*_, *P*_*Θ*_, and *P*_*loss*_, a *most parsimonious reconciliation (MPR)* for *G*^*R*^ and *S* is a DTL-scenario with minimum reconciliation cost.

Given fixed event costs, we denote the reconciliation cost of an MPR for *G*^*R*^ and *S* by *cost*(*G*^*R*^,*S*).

### Reconciliation with Unrooted Gene Trees

#### Rooting unrooted gene trees

If a gene tree is unrooted, it cannot be directly reconciled with the species tree. Thus, given an unrooted gene tree *G*^*U*^, the first step is to find a rooting for *G*^*U*^. In phylogenetic reconciliation, the standard method for rooting unrooted gene trees is to compute the reconciliation cost for each possible rooting of the unrooted gene tree and then choose a rooting that yields the minimum reconciliation cost. More formally, we choose the rooted gene tree given by $\text {arg\,min}_{{G^{R}} \in {Root}({G^{U}})} {cost}\left ({G^{R}}, S\right)$. However, there are often multiple rootings that yield the minimum reconciliation cost., i.e., that frequently $ | \text {arg\,min}_{{G^{R}} \in {Root}({G^{U}})} {cost}\left ({G^{R}}, S\right) | > 1$. In such cases, a rooted gene tree from $\text {arg\,min}_{{G^{R}} \in {Root}\left ({G^{U}}\right)} {cost}\left ({G^{R}}, S\right)$ is chosen arbitrarily for the reconciliation. For convenience, we denote the set of all optimal rootings of *G*^*R*^ with respect to *S* by *OptRoot*(*G*^*U*^,*S*), i.e., ${OptRoot}\left ({G^{U}}, S\right) = \text {arg\,min}_{{G^{R}} \in {Root}({G^{U}})} {cost}\left ({G^{R}}, S\right)$.

Reconciliation with different rootings of the same gene tree can result in drastically different reconciliations. Thus, choosing one optimal root arbitrarily when multiple optimal candidates exist can introduce many errors in the reconciliation, leading to incorrect evolutionary inferences. This source of reconciliation uncertainty is currently largely ignored in the DTL reconciliation literature and there do not exist any methods to systematically account for such uncertainty.

#### Consensus reconciliations

To properly account for rooting uncertainty, we define a *consensus reconciliation* which summarizes the different reconciliations across all optimal rootings of an unrooted gene tree and makes it possible to identify those aspects of the reconciliation that are conserved across all optimal rootings. To construct a consensus reconciliation we must first identify those subtrees in the gene tree that are conserved across all its optimal rootings, i.e., conserved across all rooted gene trees in the set *OptRoot*(*G*^*U*^,*S*). This is necessary since not all subtrees exist in all rootings of an unrooted gene tree. The set of conserved subtrees is obtained by computing the *strict consensus* [19] of all rooted gene trees in the set *OptRoot*(*G*^*U*^,*S*). For completeness, we provide the definition of strict consensus below.

##### **Definition 3**

(Strict consensus) Given a collection of rooted trees *T*_1_,*T*_2_,…,*T*_*l*_ with identical leaf sets, i.e., *Le*(*T*_1_)=*Le*(*T*_2_)=…=*Le*(*T*_*l*_), the *strict consensus* of *T*_1_,*T*_2_,…,*T*_*l*_ is a rooted tree *X* such that *Le*(*X*)=*Le*(*T*_1_)=…=*Le*(*T*_*l*_) and ${Cluster}(X) = \bigcap _{i = 1}^{l} {Cluster}(T_{i})$.

A consensus reconciliation can now be formally defined as follows:

##### **Definition 4**

(Consensus reconciliation) Given an unrooted gene tree *G*^*U*^ and a rooted species tree *S*, a consensus reconciliation for *G*^*U*^ and *S* consists of (i) the strict consensus tree ${\mathcal {SC}}$ for the trees in *OptRoot*(*G*^*U*^,*S*), (ii) for each node $g \in I({\mathcal {SC}})$ the distribution of event types (speciation, duplication, or transfer) observed for *g* across all optimal reconciliations for all gene trees in *OptRoot*(*G*^*U*^,*S*), and (iii) for each node $g \in I({\mathcal {SC}})$ the distribution of mappings (to nodes of the species tree) observed for *g* across all optimal reconciliations for all gene trees in *OptRoot*(*G*^*U*^,*S*).

The next lemma states an important and useful property of consensus reconciliations.

##### **Lemma 1**

Let ${\mathcal {SC}}$ denote the strict consensus tree of the rooted trees in *OptRoot*(*G*^*U*^,*S*). Then, each node in $I({\mathcal {SC}}) \setminus {rt}({\mathcal {SC}})$ must be binary.

##### *Proof*

Observe that the lemma follows trivially if |*OptRoot*(*G*^*U*^,*S*)|=1. Thus, in the remainder of this proof we assume that |*OptRoot*(*G*^*U*^,*S*)|≥2. Let *G*^*R*^_1_ be any optimally rooted gene tree from *OptRoot*(*G*^*U*^,*S*). Each of the other optimally rooted gene trees can be obtained by re-rooting *G*^*R*^_1_ along one of its edges. Let *A* denote the set of edges from *E*(*G*^*R*^_1_) that correspond to the other optimal rootings of *G*^*U*^. Now, define a set *B* consisting of all those edges that lie on a path between *rt*(*G*^*R*^_1_) and an edge from *A*. We label the edges in *A*∪*B* as *red* edges, and all the other edges of *E*(*G*^*R*^_1_) as *green* edges.

Consider any node *v*∈*I*(*G*^*R*^_1_) such that *E*(*G*^*R*^_1_(*v*)) contains only green edges. Since all the red edges of *G*^*R*^_1_ are outside of *G*^*R*^_1_(*v*), the subtree *G*^*R*^_1_(*v*) must appear in all the rooted gene trees from *OptRoot*(*G*^*U*^,*S*). By definition, ${Cluster}({\mathcal {SC}}) = \bigcap _{{G^{R}} \in {OptRoot}({G^{U}}, S)} {Cluster}\left ({G^{R}}\right)$, which implies that any subtree that appears in all *G*^*R*^∈*OptRoot*(*G*^*U*^,*S*) also appears in the strict consensus tree. Thus, for all nodes *v*∈*I*(*G*^*R*^_1_) such that *E*(*G*^*R*^_1_(*v*)) contains only green edges, the subtree *G*^*R*^_1_(*v*)) must appear in ${\mathcal {SC}}$. Moreover, since *v* is a binary node in *G*^*R*^_1_, it must also be binary in ${\mathcal {SC}}$.

It now suffices to show that none of the other clusters in *G*^*R*^_1_, except for the root cluster $C_{{G^{R}}_{1}}\left ({rt}\left ({G^{R}}_{1}\right)\right)$, appear in ${\mathcal {SC}}$. Consider any *u*∈*I*(*G*^*R*^_1_)∖*rt*(*G*^*R*^_1_) such that *E*(*G*^*R*^_1_(*u*)) contains a red edge. There must be at least one tree *G*^*R*^_2_∈*OptRoot*(*G*^*U*^,*S*) that is obtained by re-rooting *G*^*R*^_1_ along an edge in *E*(*G*^*R*^_1_(*u*)). Thus, the cluster $C_{{G^{R}}_{1}}(u)$ would not appear in the tree *G*^*R*^_2_. And, since ${Cluster}({\mathcal {SC}}) = \bigcap _{{G^{R}} \in {OptRoot}({G^{U}}, S)} {Cluster}\left ({G^{R}}\right)$, the cluster $C_{{G^{R}}_{1}}(u)$ cannot appear in ${\mathcal {SC}}$, as was to be shown. This implies that all non-root internal nodes of ${\mathcal {SC}}$ must be binary (corresponding to the *G*^*R*^_1_(*v*)’s with no red edges) while the root node itself must be non-binary (corresponding to the root cluster of the optimally rooted gene trees). □

Lemma 1 implies that all subtrees rooted at a non-root internal node of the strict consensus tree must, in fact, have the same topology across the different optimal rootings (i.e., that they are conserved subtrees). Observe that the consensus reconciliation shows the reconciliation for exactly those nodes that are present in the strict consensus tree. This includes the root node of the strict consensus tree, which (if non-binary) does not represent any conserved subtree and instead represents the trivial cluster representing the entire gene tree.

**Fig. 1:**
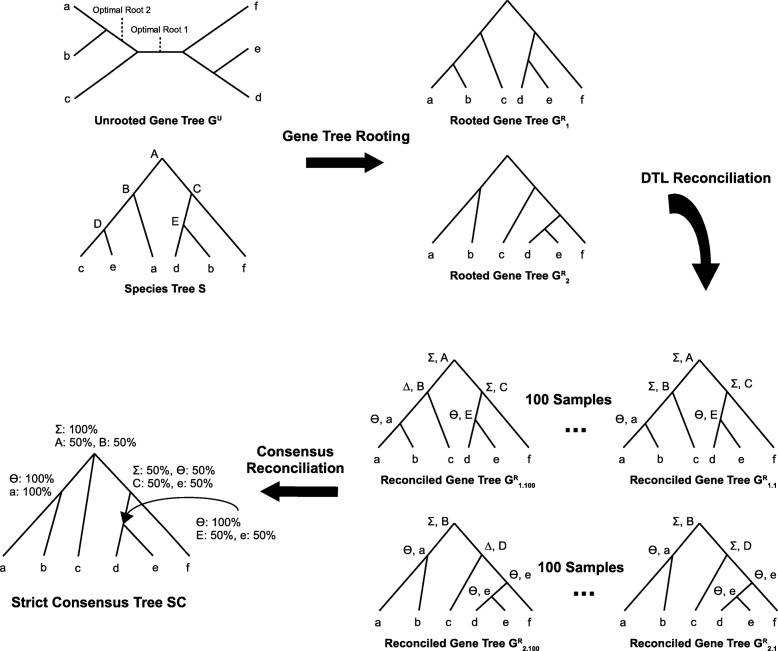
Consensus reconciliations. This figure illustrates the concept of a consensus reconciliation and shows how consensus reconciliations are computed. Given an unrooted gene tree and a species tree, the first step is to compute all optimal rootings (those that minimize the DTL reconciliation cost) of the unrooted gene tree. The second step is to reconcile each of the optimally rooted gene trees with the species tree multiple times to sample the space of all most parsimonious reconciliations uniformly at random; this sampling is required to account for any variation in different most parsimonious reconciliations for the same optimally rooted gene tree. In the figure, *Σ*,*Δ*, and *Θ* denote speciation, duplication, and transfer events, respectively. Each internal node in the reconciled tree is labeled with both its event type and the species tree node to which it maps. The final step is to aggregate each of the computed reconciliations into a single consensus reconciliation that shows the reconciliation of all those portions of the gene tree that are conserved across all optimal rootings. Thus, the tree underlying the consensus reconciliation is the strict consensus tree of all optimal rootings. Each internal node of this strict consensus tree is labeled with aggregated reconciliation information for that node from all sampled reconciliations across all optimal rootings

In constructing a consensus reconciliation one must account for the fact that even a rooted gene tree may have many different optimal DTL reconciliations. To account for this additional source of reconciliation uncertainty, we make use of standard techniques for handling multiple optimal reconciliations. Specifically, for each optimal rooting of the gene tree, we sample the space of optimal reconciliations uniformly at random [8], computing 100 such samples for each rooting. We then compute, for each node in the strict consensus tree, an aggregation of the mapping and event assignments for that node across all different optimal rootings and all sampled reconciliations for each rooting. Figure 1 illustrates the concept and construction of consensus reconciliations.

#### Maximum size of a consensus reconciliation

The number of internal nodes in a strict consensus tree on *n* leaves can range between 1 and *n*−1, depending on how many clusters appear in the strict consensus tree. We refer to the number of internal nodes in the strict consensus tree of all optimal rootings of a gene tree as the *size* of that strict consensus tree. The size of such a strict consensus tree depends on three factors: First, the number of leaves, say *n*, in the unrooted gene tree. Second, the number of multiple optimal rootings, say *k*, for that gene tree. And third, the placement of these optimal rootings on the unrooted gene tree.

The next lemma provides a tight upper bound on the size of the strict consensus tree for any fixed value of *n* and *k*.

##### **Lemma 2**

Given an unrooted gene tree *G*^*U*^ with *n* leaves and *k* distinct optimal rootings, the strict consensus tree ${\mathcal {SC}}$ for the trees in *OptRoot*(*G*^*U*^,*S*) can have no more than $(n - 1) - \left \lfloor \frac {k}{2} \right \rfloor $ internal nodes. Furthermore, there exists a placement of the *k* roots on *G*^*U*^ such that ${\mathcal {SC}}$ has exactly $(n -1) - \left \lfloor \frac {k}{2} \right \rfloor $ internal nodes.

##### *Proof*

Observe that, since the number of internal nodes in any rooted binary tree with *n* leaves is *n*−1, the lemma is trivially correct if *k*=1. Let *G*^*R*^_1_ be any optimally rooted gene tree from *OptRoot*(*G*^*U*^,*S*). We partition the edges of *G*^*R*^_1_ into *red* and *green* edges exactly as described in the proof of Lemma 1. Let *r* denote the number of nodes *v*∈*I*(*G*^*R*^_1_)∖*rt*(*G*^*R*^_1_) that contain a red edge. From the proof of Lemma 1 we know that if *v*∈*I*(*G*^*R*^_1_) is such that *E*(*G*^*R*^_1_(*v*)) contains only green edges, then the subtree *G*^*R*^_1_(*v*)) must appear in ${\mathcal {SC}}$, and that if *v*∈*I*(*G*^*R*^_1_)∖*rt*(*G*^*R*^_1_) is such that *E*(*G*^*R*^_1_(*v*)) contains a red edge then the cluster $C_{{G^{R}}_{1}}(v)$ cannot appear in ${\mathcal {SC}}$. This implies that the number of internal nodes in ${\mathcal {SC}}$ must be exactly equal to (*n*−1)−*r*. It now suffices to show that $\left \lfloor \frac {k}{2} \right \rfloor $ is a tight lower bound on the value of *r*.

Consider a placement of the remaining *k*−1 roots along the edges of *G*^*R*^_1_ in a level-by-level breadth-first traversal starting at the level immediately below the edges incident on the root of *G*^*R*^_1_. The key observation is that, with such a placement, the size of *r* increases by exactly one for every two additional roots placed on *G*^*R*^_1_ (since each internal node of the tree has exactly two child-edges, the placement of a root on one or both of which affects only that internal node and nothing else). More precisely, if an even number of additional roots have been placed, then the placement of the next root will increase the value of *r* by 1, while if an odd number of additional roots have been placed, then adding the next root will not affect any new internal nodes and therefore leave *r* unchanged. This placement thus corresponds to a value of $\left \lfloor \frac {k}{2} \right \rfloor $ for *r*. Moreover, a placement of the *k*−1 additional roots for which $r < \left \lfloor \frac {k}{2} \right \rfloor $ is only possible if at least one of the internal nodes of *G*^*R*^_1_ has more than two children. Hence, the level-by-level breadth-first placement must be optimal, showing that $\left \lfloor \frac {k}{2} \right \rfloor $ is a tight lower bound on *r*. □

Lemma 2 will be useful later for estimating how “closely” the set of optimal rootings is clustered together on its gene tree. It will also be useful for comparing the actual size (or information content) of the consensus reconciliation for a gene tree against the maximum possible size of a consensus reconciliation for that gene tree. We refer to optimal rootings that are clustered as closely as possible (thus maximizing the size of the consensus reconciliation) as *maximally clustered* optimal rootings. More formally:

##### **Definition 5**

(Maximally clustered rootings) Given an unrooted gene tree *G*^*U*^ with *n* leaves and *k* distinct optimal rootings, we say that the *k* optimal rootings are *maximally clustered* if the strict consensus tree ${\mathcal {SC}}$ for the trees in *OptRoot*(*G*^*U*^,*S*) has exactly $(n - 1) - \left \lfloor \frac {k}{2} \right \rfloor $ internal nodes.

**Fig. 2:**
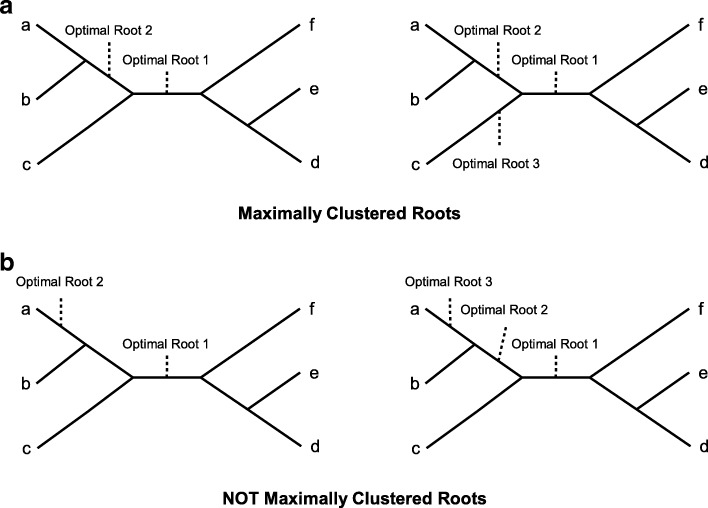
Maximal clusterings. This figure illustrates the concept of maximal clustering of optimal roots on a gene tree. The unrooted gene trees in (**a**) have maximally clustered optimal roots such that those roots are as close together on the tree as possible. On the other hand, the gene trees in (**b**) do not have maximally clustered optimal roots since those roots are not as close together as possible on either of those trees

Figure 2 illustrates the concept of maximally clustered optimal rootings on a gene tree.

## Results and discussion

### Description of the data set and experimental setup

For our analysis we used a biological data set of over 4700 gene families from a broadly sampled set of 100, predominantly prokaryotic, species [4]. We constructed two sets of gene trees for the gene families in the data set. The first set was constructed using RAxML [20], a standard and widely used software package for constructing maximum likelihood trees. In the interest of time, we terminated runs that took longer than two days (gene trees with many hundreds of leaves), resulting in 4571 RAxML gene trees. The second set of gene trees was constructed using the gene tree error correction software TreeFix-DTL [21], and these TreeFix-DTL trees represent error-corrected versions of the RAxML trees. We again terminated runs taking longer than a few days of running time, resulting in 4547 TreeFix-DTL gene trees. Our set of RAxML gene trees represents a “default” set of gene trees constructed using a standard, commonly used method for gene tree construction, while the set of TreeFix-DTL trees represents a more accurate set of gene trees with fewer topological errors [21] constructed using a state-of-the-art error-correction method. Analyzing these two sets of gene trees separately makes it possible to assess the impact of gene tree error on the prevalence and structure of multiple optimal rootings.

For computing DTL reconciliations, we used a default event cost assignment of 〈1,2,3〉 for loss, duplication, and transfer events, respectively, as well as two additional cost assignments 〈1,2,2〉 and 〈1,2,5〉 to study the impact of low and high transfer costs on the prevalence of multiple optimal rootings.

Finally, to assess the impact of using a dated species tree on multiple optimal roots, we also used a dated species tree and restricted transfer events to only occur between coexisting species [5].

### Prevalence of optimal rootings

**Fig. 3:**
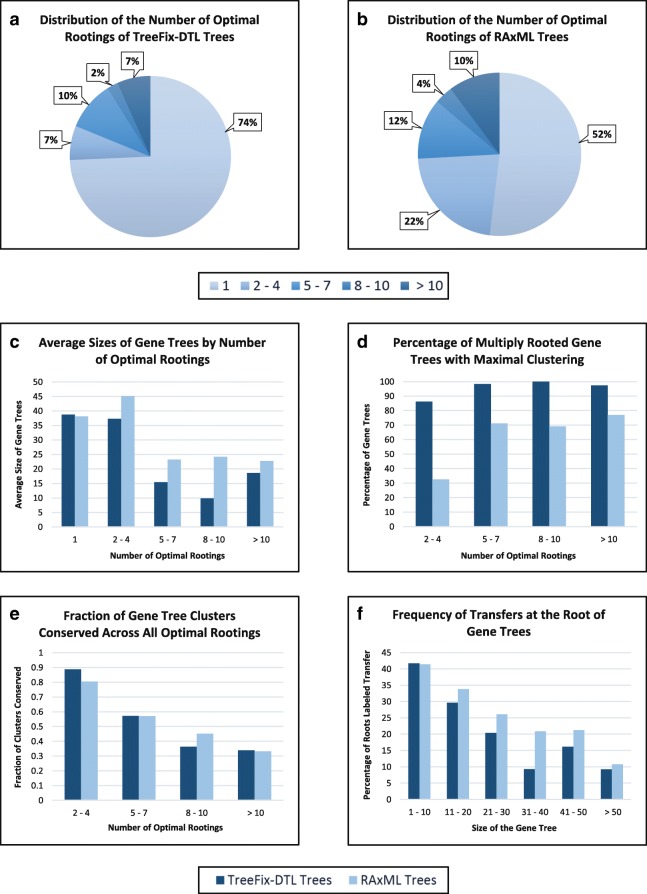
Experimental results. **a** and **b** Fraction of gene trees in the data set with the specified number of optimal rootings, for the TreeFix-DTL and RAxML gene trees, respectively. **c** Average gene tree size, in terms of number of leaves, for the TreeFix-DTL and RAxML trees, for gene trees with different numbers of optimal rootings. **d** Percentage of multiply rooted gene trees that have maximally clustered rootings for different numbers of optimal rootings. **e** Fraction of gene tree clusters conserved across all optimal rootings, for different numbers of optimal rootings. **f** Relationship between gene tree size and frequency of transfer events at their roots. Results shown are based on DTL reconciliation with loss, duplication, and transfer costs of 1, 2, and 3, respectively, and with an undated species tree. Gene tree sizes are shown in terms of number of leaves

**Basic results and impact of gene tree error.** We computed all optimal rootings for our two collections of gene trees (RAxML trees and TreeFix-DTL trees) using the standard event cost assignment of 〈1,2,3〉 for loss, duplication, and transfer events, respectively, and using an undated species tree for reconciliation. The number of gene trees with multiple optimal rootings varied widely across the two collections of gene trees. Specifically, 2197 of the 4571 RAxML gene trees had more than one optimal root, while only 1168 of the 4547 TreeFix-DTL gene trees had more than one optimal root. This dramatic difference of 48.1% of gene trees for RAxML vs 25.7% of gene trees for TreeFix-DTL is due to the higher topological error rate in the RAxML gene trees, and suggests that error in gene trees can greatly inflate the number of optimal rootings. Furthermore, the fact that over a quarter of the relatively accurate TreeFix-DTL gene trees have multiple optimal roots shows that ambiguous rooting assignment is a significant problem in practice even when using accurate gene trees. We also measured the average number of optimal rootings across the gene trees with multiple optimal roots: The 2197 RAxML gene trees had, on average, 7.3 optimal roots, while the 1168 TreeFix-DTL gene trees had 8.2. Parts (a) and (b) of Fig. 3 show the distribution of the number of optimal rootings for the TreeFix-DTL and RAxML gene trees.

**Relationship to gene tree size.** Next, we calculated the average sizes of the gene trees, in terms of their number of leaves, with one and with multiple optimal rootings. Surprisingly, we found that the gene trees with more than one optimal root are significantly smaller than the gene trees with only one optimal root. Specifically, for the TreeFix-DTL gene trees, the average size of gene trees with multiple roots is 21.6, while for the rest of the gene trees it is 38.7. The difference is less dramatic for the RAxML gene trees, with average sizes 33.3 and 38.1, respectively, but this is likely due to the high error rate of RAxML trees and the corresponding inflation in the number of gene trees with multiple optima. Figure 3c shows the average sizes of gene trees with different numbers of optimal rootings. Overall, this analysis suggests that multiple optimal roots are more common when smaller gene trees are reconciled with a larger species tree. Larger gene trees, with genes from a larger fraction of the species represented in the species tree, are perhaps more likely to have sufficient topological information to have only a single root with minimum reconciliation cost.

In the remainder of this section, we report detailed results only for the more accurate TreeFix-DTL gene trees. In general, we observed that the same overall patterns also held for the RAxML gene trees.

**Impact of using different transfer costs.** We repeated the above analysis twice, using transfer costs 2 and 5 (and keeping other event costs the same). A transfer cost of 2 implies that many more transfer events are inferred, while a transfer cost of 5 leads to fewer transfer events being invoked. For the TreeFix-DTL trees, using a transfer cost of 2, the number of trees with multiple optimal roots and the average number of optimal roots per multiply rooted gene tree both increase significantly to 2343 and 12.6, respectively. With a transfer cost of 5, the corresponding values decrease to 1014 and 6.2, respectively, for the TreeFix-DTL trees. A similar pattern of increase and decrease was observed when using transfer costs 2 and 5, respectively, for the RAxML trees. These results suggest that the prevalence of multiple optimal roots is positively correlated with the number of inferred transfer events.

**Impact of using dated species tree.** To understand the effect of using a dated species tree, we used a dated version of the same species tree (obtained from [4]) and restricted transfer events to only occur between coexisting species using the dated DTL reconciliation model described in [5]. For the TreeFix-DTL trees, we observed that the number of gene trees with multiple optimal roots increased to 1561, compared to 1168 with the undated species tree. However, the average number of optimal rootings across the gene trees with multiple optimal roots decreased to 5.5, compared to 8.1 with the undated species tree. Thus, even though there were more trees with multiple roots, the number of optimal roots per gene tree decreased. For the RAxML gene trees, the number of gene trees with multiple roots stayed almost unchanged, likely since that number is already inflated even when using the undated species tree, while the average number of optimal rootings showed the same decreasing trend as the TreeFix-DTL trees and reduced from 7.3 with the undated species tree to 5.1 for the dated species tree.

### Structure of optimal rootings

**Arrangement of optimal roots on gene trees.** We analyzed the gene trees that had multiple optimal roots and studied the arrangement of their optimal root positions. We first used the result of Lemma 2 to compute the number of gene trees that had maximally clustered optimal rootings. Of the 1168 TreeFix-DTL gene trees with multiple roots, we found that 1110, i.e., 95%, had maximally clustered rootings. Thus, for the vast majority of the multiply rooted gene trees, all optimal roots were clustered closely together on the gene tree. This is a highly desirable property since it makes it easier to estimate the “true” root position and also maximizes the size of the consensus reconciliation, leading to more complete evolutionary inferences even after accounting for rooting uncertainty. Figure 3d shows how the fraction of gene trees with maximally clustered rootings varies as the number of optimal rootings increases. Interestingly, we observed a striking difference between the average sizes of the multiply rooted gene trees with maximally clustered rootings and those without, with average sizes 19.8 and 57.1, respectively. In line with the previous observation that smaller gene trees tend to have more optimal rootings, the average number of optimal rootings is significantly higher for the gene trees that are maximally clustered (8.3) versus the gene trees that do not have maximally clustered rootings (4.7). For the RAxML gene trees, we found that a much smaller fraction of multiply rooted gene trees had maximally clustered rootings, only 1197 out of 2197, pointing again to the very large impact of gene tree error on the structure of optimal rootings.

We further studied those gene trees whose optimal roots were not maximally clustered to gauge how clustered together the roots were in this case. We computed consensus reconciliations and calculated, for each such gene tree, the number of internal nodes in its consensus reconciliation and divided this by the theoretically maximum possible size for that consensus reconciliation based on Lemma 2. We call this ratio the *clustering ratio*. The more clustered the optimal roots of a gene tree, the closer this ratio is to 1, while a less clustered set of rootings pushes the ratio towards 0. The average clustering ratio was 0.88 for the TreeFix-DTL gene trees whose roots were not maximally clustered. This clustering ratio is close to 1, suggesting that even when optimal roots are not maximally clustered, they tend to be close to each other on the gene tree. Results were similar for the RAxML trees, with a clustering ratio of 0.79 for gene trees whose roots were not maximally clustered.

**Interesting patterns for singly rooted and multiply rooted gene trees.** When comparing singly rooted and multiply rooted TreeFix-DTL gene trees, we noticed that the roots of singly rooted gene trees are predominantly (95% of the time) labeled as speciation events and were never labeled as a transfer event, while the roots of multiply rooted gene trees had a much more equitable distribution of assigned event types with 37.6*%* of the roots labeled as speciations, 22.3*%* as duplications, and 40.1*%* as transfers. This is a surprising result and suggests that the presence of a transfer at the root is a very strong indication of the presence of multiple optimal roots. We also noticed that smaller gene trees are far more likely to have transfer events at their roots. This relationship is clearly depicted in Fig. 3f, and holds true for both TreeFix-DTL and RAxML trees. This observation also helps explain the previously discussed relationship between gene tree size and prevalence of multiple optimal rootings where we observed that smaller gene trees tend to have more optimal rootings.

When considering only multiply rooted TreeFix-DTL gene trees, we observed that multiply rooted gene trees without maximally clustered rootings had almost 71% of root nodes labeled as speciations compared to only 37% for the multiply rooted gene trees with maximally clustered rootings. This may be partly due to the fact that the gene trees that do not have maximally clustered rootings are significantly larger on average and thus have significantly fewer transfer events at their roots.

### Consensus reconciliations

**Size of consensus reconciliations.** Next, we analyzed the consensus reconciliation for each multiply rooted TreeFix-DTL gene tree and measured the sizes of the consensus reconciliations. Recall that a consensus reconciliation only shows the reconciliation for those portions of the gene tree that are conserved across all its optimal rootings. Thus, we first measured how much of the gene tree is actually conserved across all rootings, i.e., for each unrooted gene tree, we calculated the number of internal nodes in the strict consensus of its optimal rootings divided by the number of internal nodes in any one of the optimal rootings. This is motivated by the simple observation that a larger consensus reconciliation contains more evolutionary information about the original unrooted gene tree than a smaller consensus reconciliation for that tree. For all TreeFix-DTL gene trees, this ratio was 0.89, showing that across the entire data set, reconciliation information could be inferred for 89% of the nodes in the gene trees even after accounting for multiple optimal rootings. When limiting this analysis to only multiply rooted gene trees, the ratio falls to 0.58, indicating that even for multiply rooted gene trees, reconciliation information can be meaningfully inferred for almost 60% of the nodes in the gene tree. This ratio is not any larger simply because of the small average size of the multiply rooted gene trees and the large average number of optimal rootings in those trees. Figure 3e shows this ratio for multiply rooted gene trees with different numbers of optimal roots for both TreeFix-DTL and RAxML trees.

**Event and mapping inference from consensus reconciliations.** We checked how often the nodes of the consensus reconciliation were assigned a fully consistent event type or mapping across all optimal rootings and all sampled optimal reconciliations for each rooting. (Recall that to account for reconciliation uncertainty, in addition to rooting uncertainty, we randomly sample 100 optimal reconciliations for each optimal rooting.) We observed that 93% of the nodes in the consensus reconciliations of the multiply rooted TreeFix-DTL trees were assigned a consistent event type (speciation, duplication, or transfer), while 83% were mapped consistently to the same node on the species tree. These numbers are only about 5% smaller than the averages for singly rooted gene trees, showing that the event and mapping assignments remain overwhelmingly conserved across different optimal rootings. Overall, these results show that unrooted gene trees can be meaningfully reconciled and high-quality evolutionary information can be obtained from them even after accounting for multiple optimal rootings. Corresponding numbers for the RAxML trees were 89 and 67%, respectively, showing that gene tree error greatly affects not only the prevalence and structure of optimal rootings but also the consistency of event and mapping assignments in the reconciliation itself.

Surprisingly, we observed that the root nodes of consensus reconciliations (of multiply rooted gene trees) had very low event and mapping consistency compared to other nodes in consensus reconciliations. Specifically, for the multiply rooted TreeFix-DTL trees, only 11% of the root nodes had a consistently assigned event and only 5% had a consistently assigned mapping. For the RAxML trees, these numbers were 36 and 8%, respectively. This is in stark contrast to the very high consistency of events and mappings for the non-root nodes in the consensus reconciliations (98 and 88%, respectively, for TreeFix-DTL trees, and 91 and 70%, respectively, for RAxML trees). In addition, and also to our surprise, we observed that each of the 3379 singly rooted TreeFix-DTL trees and 2373 of the 2374 singly rooted RAxML trees had a consistent mapping and event assignment at the root. This, again, stands in stark contrast to the root mapping and event assignments for multiply rooted gene trees. These observations have important implications for studies focused on inferring locations of gene birth on the species tree, e.g. [4], especially when gene tree rooting is uncertain.

## Conclusion

In this paper, we studied the problem of DTL reconciliation with unrooted gene trees. We provided the first in-depth analysis of the prevalence and structure of multiple optimal rootings and of their impact on the inferred reconciliation. We introduced the notion of a consensus reconciliation, which accounts for rooting uncertainty, and provide the first computational tools for computing consensus reconciliations. Our analysis uncovered the drastic impact of gene tree error on optimal rootings, and we also studied the impact of alternative event cost assignments and of using dated DTL reconciliation. Our results confirm that a significant fraction of gene trees that are used for DTL reconciliation have multiple optimal rootings. They also show that the number of these optimal roots is especially high for trees that are smaller in size. However, since most of these optimal roots are closely clustered together in the gene tree, we discovered that the number of subtrees in the gene tree actually affected by the presence of multiple optimal roots is relatively low. Furthermore, we found that the vast majority of the subtrees that are conserved across all of the optimal rootings of a gene tree are reconciled identically across all optimal rootings. Our results, along with the new computational tools and techniques introduced in this paper, will help biologists perform more accurate analysis of gene family evolution by explicitly accounting for uncertainty in gene tree rooting when using DTL reconciliation.

This work provides several useful directions for future research. For instance, it would be useful to investigate if the fact that optimal roots almost always appear clustered together on any gene tree can be used to estimate the “true” root for that gene tree. Similarly, it would be interesting and informative to systematically compare the accuracy of gene tree rooting using DTL reconciliation to other rooting methods and to identify the evolutionary conditions under which reconciliation-based rooting fails to perform well.

## References

[1] Gorbunov KY, Liubetskii VA (2009). Reconstructing genes evolution along a species tree. Mol Biol.

[2] Doyon JP, Scornavacca C, Gorbunov KY, Szöllosi GJ, Ranwez V, Berry V, Tannier E (2010). An Efficient Algorithm for Gene/Species Trees Parsimonious Reconciliation with Losses, Duplications and Transfers. RECOMB-CG. vol. 6398 of Lecture Notes in Computer Science.

[3] Tofigh A, Hallett MT, Lagergren J (2011). Simultaneous Identification of Duplications and Lateral Gene Transfers. IEEE/ACM Trans Comput Biol Bioinform.

[4] David LA, Alm EJ (2011). Rapid evolutionary innovation during an Archaean genetic expansion. Nature.

[5] Bansal MS, Alm EJ, Kellis M (2012). Efficient algorithms for the reconciliation problem with gene duplication, horizontal transfer and loss. Bioinformatics.

[6] Stolzer M, Lai H, Xu M, Sathaye D, Vernot B, Durand D (2012). Inferring duplications, losses, transfers and incomplete lineage sorting with nonbinary species trees. Bioinformatics.

[7] Szollosi GJ, Boussau B, Abby SS, Tannier E, Daubin V (2012). Phylogenetic modeling of lateral gene transfer reconstructs the pattern and relative timing of speciations. Proc Natl Acad Sci.

[8] Bansal MS, Alm EJ, Kellis M (2013). Reconciliation Revisited: Handling Multiple Optima when Reconciling with Duplication, Transfer, and Loss. J Comput Biol.

[9] Scornavacca C, Paprotny W, Berry V, Ranwez V (2013). Representing a set of reconciliations in a compact way. J Bioinform Comput Biol.

[10] Libeskind-Hadas R, Wu YC, Bansal MS, Kellis M (2014). Pareto-optimal phylogenetic tree reconciliation. Bioinformatics.

[11] Sjostrand J, Tofigh A, Daubin V, Arvestad L, Sennblad B, Lagergren J (2014). A Bayesian Method for Analyzing Lateral Gene Transfer. Syst Biol.

[12] Kordi M, Bansal MS (2016). Exact Algorithms for Duplication-Transfer-Loss Reconciliation with Non-Binary Gene Trees. Proceedings of the 7th ACM International Conference on Bioinformatics, Computational Biology, and Health Informatics, BCB 2016, Seattle, WA, USA, October 2-5 2016.

[13] Jacox E, Chauve C, Szollosi GJ, Ponty Y, Scornavacca C (2016). ecceTERA: comprehensive gene tree-species tree reconciliation using parsimony. Bioinformatics.

[14] Ovadia Y, Fielder D, Conow C, Libeskind-Hadas R (2011). The Cophylogeny Reconstruction Problem Is NP-Complete. J Comput Biol.

[15] Libeskind-Hadas R, Charleston M (2009). On the Computational Complexity of the Reticulate Cophylogeny Reconstruction Problem. J Comput Biol.

[16] Rutschmann F (2006). Molecular dating of phylogenetic trees: A brief review of current methods that estimate divergence times. Divers Distrib.

[17] Urbini Laura, Sinaimeri Blerina, Matias Catherine, Sagot Marie-France (2016). Robustness of the Parsimonious Reconciliation Method in Cophylogeny. Algorithms for Computational Biology.

[18] Górecki P, Eulenstein O, Tiuryn J (2013). Unrooted Tree Reconciliation: A Unified Approach. IEEE/ACM Trans Comput Biol Bioinform.

[19] McMorris FR, Meronk DB, Neumann DA, Felsenstein J (1983). A View of Some Consensus Methods for Trees. Numerical Taxonomy.

[20] Stamatakis A (2006). RAxML-VI-HPC: maximum likelihood-based phylogenetic analyses with thousands of taxa and mixed models. Bioinformatics.

[21] Bansal MS, Wu YC, Alm EJ, Kellis M (2015). Improved gene tree error correction in the presence of horizontal gene transfer. Bioinformatics.

